# Diversity and Structure of the Prokaryotic Community in Tropical Monomictic Reservoir

**DOI:** 10.1007/s00248-025-02508-1

**Published:** 2025-03-12

**Authors:** Mariel Barjau-Aguilar, Ana M. J. Reyes-Hernández, Martín Merino-Ibarra, Gloria Vilaclara, Jorge Alberto Ramírez-Zierold, Rocío J. Alcántara-Hernández

**Affiliations:** 1 Instituto de Geología, Universidad Nacional Autónoma de México, Ciudad Universitaria, Av. Universidad 3000, Del. Coyoacán, 04510 Ciudad de Mexico, México; 2 Posgrado en Ciencias Biológicas, Universidad Nacional Autónoma de México, Unidad de Posgrado, Edificio D, 1° Piso, Circuito de Posgrados, Ciudad Universitaria, Coyoacán, 04510 Ciudad de Mexico, México; 3 Unidad Académica de Biodiversidad Acuática, Instituto de Ciencias del Mar y Limnología, Universidad Nacional Autónoma de México, Av. Universidad 3000, Ciudad Universitaria Coyoacán, C.P. 04510 Ciudad de Mexico, México; 4 Grupo de Investigación en Limnología Tropical, FES Iztacala, Universidad Nacional Autónoma de México, Tlalnepantla, 54090 Estado de México México

**Keywords:** 16S rRNA gene, Bacteria, Water reservoir, Eutrophic, Nutrient, Stratification-circulation

## Abstract

Bacteria and Archaea are microorganisms that play key roles in the biogeochemical transformations that control water quality in freshwater ecosystems, such as in reservoirs. In this study, we characterize the prokaryotic community of a high-relevance tropical eutrophic reservoir using a 16S rRNA gene survey during a low-water level fluctuation period mainly used for storage, associating the distribution of these microorganisms with the hydrogeochemical conditions of the water column. Our findings revealed that diversity and structure of the prokaryotic community exhibited spatio-temporal variations driven by the annual circulation-stratification hydrodynamic cycle and are significantly correlated with the concentrations of dissolved oxygen (DO), soluble reactive phosphorus (SRP), and dissolved inorganic nitrogen (DIN). During the heterotrophic circulation, the breakdown of thermal gradient leads to a homogeneous distribution of the nutrients, where the presence of DO promotes the dominance of aerobic and facultative heterotrophic bacteria such as *Bacteroidota*, *Actinobacteriota*, and *Verrucomicrobiota.* Also, the autotrophic circulation was characterized by an increase of DO and NO_3_^−^ concentrations, with abundant *Cyanobacteria*. Finally, during the stratification, the presence of prokaryotes associated with the metabolism of CH_4_ was detected, mainly in the hypolimnion, as well as others related to sulfate reduction and nitrification. This study shows the diversity of the prokaryotic community in tropical eutrophic reservoirs, and how the continuous monitoring with metabarcoding techniques can provide critical insights for a deeper understanding of the biogeochemical dynamics and improve the water resource management in the future.

**Supplementary Information:**

The online version contains supplementary material available at 10.1007/s00248-025-02508-1.

## Introduction

Water supplies around the world are being threatened by population growth, anthropic activities, and global change, negatively affecting human development [1, 2]. Additionally, industrial and economic activities have compromised water quality in surface water bodies, primarily due to eutrophication processes that promote changes in the bacterial community and in the biogeochemical dynamics of the aquatic systems [3–5]. One partial solution has been the construction of reservoirs in populated areas to increase water availability. By the end of the twenty-first century, it is expected that the number of reservoirs worldwide will be ten times higher than in 1950 [6]. However, ensuring the water supply also requires well-structured management policies that prioritize in the long-term, the health of the ecosystems, water quality, and safety. To achieve these goals, it is essential to understand the hydrodynamics and biogeochemical processes of the nutrients in epicontinental aquatic systems, such as carbon (C), nitrogen (N), and phosphorous (P) [3, 7–9], where microorganisms drive several transformation processes.

Microbial communities play a crucial role in biogeochemical cycles at global and ecosystemic scale, including all aquatic ecosystems [10–12]. In recent decades, the development of culture-independent methods, such as metabarcoding studies, has enabled us to characterize the entire prokaryotic community [13], where the study of aquatic ecosystems can be done with a relatively minimal collecting and processing efforts, and in a relatively short timeframe [14, 15]. Microbial survey coupled with a spatio-temporal monitoring approach in water bodies are key for the tracking of water quality and environmental health [13]. In the long term, the integration of the metabarcoding analysis with the physicochemical and biogeochemical characterization of the water column could establish a powerful approach to better understand the biogeochemical transformations in key aquatic systems, such as reservoirs. Furthermore, the ecosystemic services that microbiota provide to reservoirs is valuable, such as the oxidation of methane, which has been found as a critical process linked to the microbial transformation in the C, N, and S cycles [16]. Because of this, studying the prokaryotic communities of reservoirs overtime is critical for assessing the impacts of anthropic activities on key processes such as the attenuation of greenhouse gases (also known as GHGs).

**Fig. 1:**
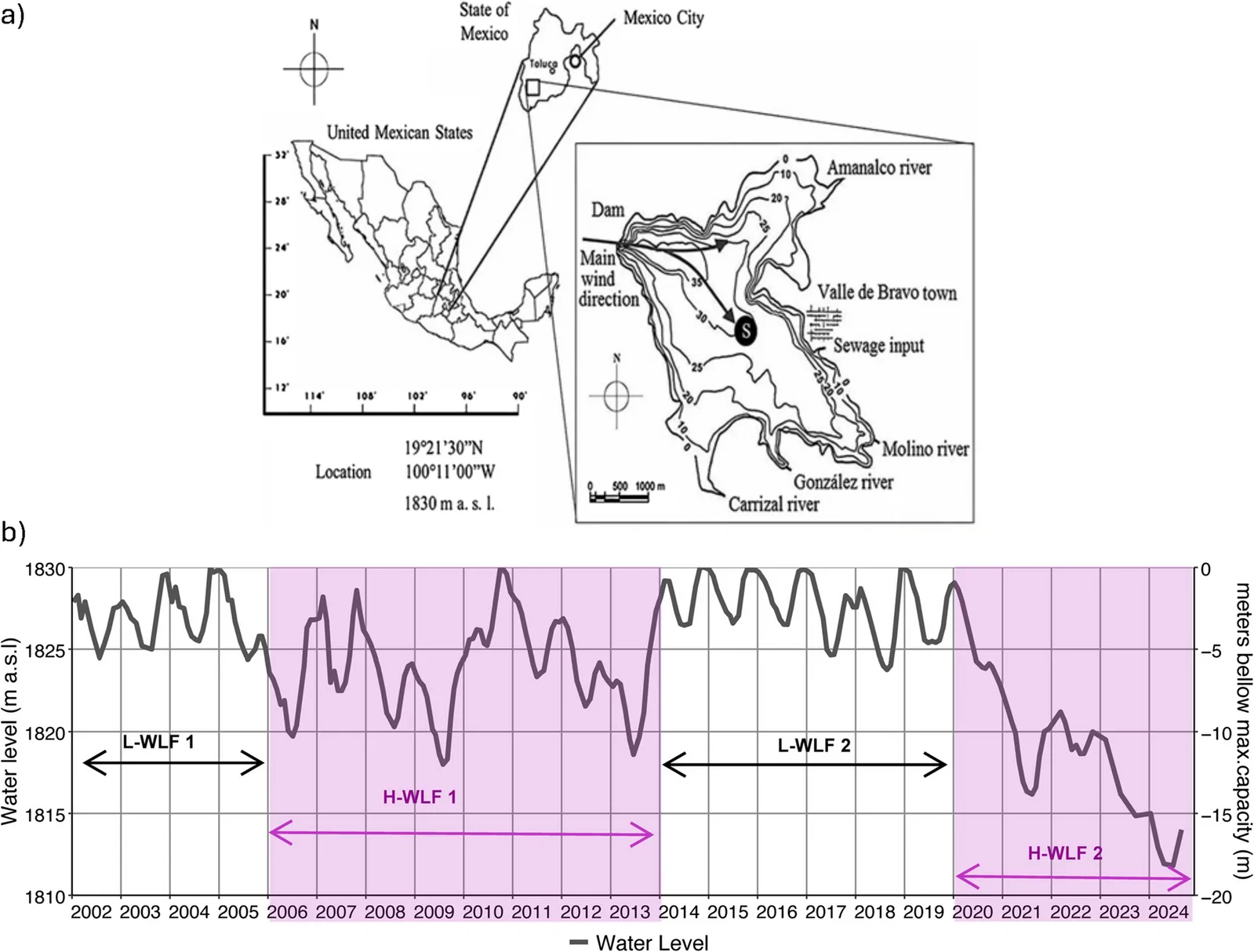
The reservoir of Valle de Bravo. **a**) Bathymetry of the Reservoir of Valle de Bravo with the location of the central monitoring station, all the inputs (rivers and sewages) and the water withdrawal through pump-out at the dam. Depth contours are reported in meters below the maximum level of the reservoir, adapted Merino-Ibarra et al. [29] and Valeriano-Riveros et al. [23]. **b**) Water level fluctuations of the reservoir of Valle de Bravo from 2002 to 2024, adapted from Barjau-Aguilar et al. [22]. The left axis shows the water level in meters above sea level, while the right axis displays level in meters (m) below its maximum capacity (1830 m a.s.l.). Periods of Low Water-Level Fluctuations periods (L-WLF) are indicated with black arrows, and periods of High Water-Level Fluctuations (H-HWLF) are marked with purple arrows and shaded areas. The study period of this work (December 2018 to January 2020) is depicted with a blue box

In reservoirs, water level fluctuations are induced by the anthropic management that includes the extraction and injection of large amounts of water, changing its hydrothermal pattern, the concentration and dynamics of nutrients in the water column, and consequently, its microbiology [17–19]. Furthermore, tropical aquatic systems differ from their temperate counterparts in their thermal gradients in the water column, due to environmental factors such as solar radiation, among others [20, 21]. To date, there is limited information regarding the microbial composition in these man-made aquatic ecosystems. Therefore, the aim of this study was to characterize the prokaryotic community in one of the most long-term studied tropical reservoirs in Mexico, the one of Valle de Bravo (VB), through an entire hydrodynamic cycle during a low-water level fluctuation period (L-WLF) [22], and to associate the distribution of these microorganisms with the hydrogeochemical conditions of the water column. Since July 2013, water injections were done in VB to increase the water level, reaching 72 to 100% of its maximum capacity until the beginning of 2020. This has been a historical key point of maximum level with low water level fluctuations (Fig. 1), that can be considered as a baseline condition for the reservoir. Despite the relevance of the reservoir, there has not been reports including the survey of the prokaryotic community in the system, and only partial visions have been reported such as the taxonomic composition of the phytoplankton [23] or the seasonal variations of the zooplankton [24].

This research seeks to draw a molecular baseline to identify the prokaryotic community in this relevant aquatic system, and its changes during a hydrodynamic period with small changes in the water level, to assess the relationships between physicochemical and biogeochemical parameters, and the structure and composition of the prokaryotic community in these baseline conditions (i.e., in L-WLF conditions and near the reservoir’s maximum capacity), as well as to have a perspective of its potential role in the biogeochemical processes. We look forward that our findings will be useful for environmental policymakers of tropical reservoirs such as VB, which are expected to increase in numbers in these latitudes [6, 25]. This work can help in the future to enhance the identification of scenarios where water quality may be compromised and provide recommendations to local authorities.

## Material and Methods

### Study Site

The reservoir of Valle de Bravo (VB) (19°21′30′′ N, 100°11′00′′ W) (Fig. 1a) supplies approximately 20–25% of the water consumed in Mexico City [26], which is the fourth largest megalopolis in the world. VB is the largest reservoir of the Cutzamala System, which represents one of the highest investments in the water supply infrastructure of Mexico, transporting water over 127 km and elevating it over 1,100 m from the western region of the country into Mexico City, and the Valley of Toluca [19]. In addition, VB is vital for the local economy, which mainly relies on tourism, cultural and recreational activities, such as sailing [26]. Olvera-Viascán et al. [27] suggested that in 1992 the reservoir was in a eutrophic state due to the arriving loads of P; since, other studies have suggested a eutrophic condition based on the P and N loads [19, 22] AND the C ecosystemic metabolism [28], a trophic condition that is found nowadays.

From the limnological point of view, VB is a warm monomictic water body, where circulation occurs during winter months (October to February) and stratification during the rest of the year (March to September). This thermal regime shapes the evolution of physicochemical and biogeochemical parameters in both, time and spatial scales throughout the water column [28, 29]. Since 1992, eutrophication of VB is a matter of concern for local users and Mexico City inhabitants, because of its public health and environmental implications [27]. Furthermore, the increasing water demand of the megalopolis has added impact on this aquatic system, as well as the extension of dry seasons in recent years [22, 30]. As a result, VB has experienced periods of high water-level fluctuations (H-WLF) –and periods of low water-level fluctuations (L-WLF), when the level was above its historical mean and the main purpose of the reservoir was to store water (Fig. 1b). These water level fluctuations have important effects on the biogeochemical dynamics and may directly impact water quality [22, 30].

To address this critical situation, our multidisciplinary research group has been monitoring VB since 2001, exploring various topics that include: limnological characterization [29], the impact of water level fluctuations in the vertical boundary mixing process [30], the ecosystem metabolism [28, 31], the N and P biogeochemical dynamics [19, 22] and the taxonomical characterization of the phytoplanktonic community [23]. However, research has not focused yet on the prokaryotic community, which are the key players in biogeochemical cycles.

To address the study of the prokaryotic community of VB for the first time, we implemented a spatio-temporal sampling program for metabarcoding analysis in 2018, covering an annual hydrodynamic cycle. Our goal was to characterize these communities and understand their relationship with the physicochemical and biogeochemical conditions that occur associated to the monomictic behavior that has been documented for VB during L-WLF periods (specifically in this case during L-WLF2, Fig. 1b), to establishing a prokaryotic molecular baseline for future studies.

### Sampling

Samples were collected in a spatio-temporal scale, considering the epilimnion, metalimnion, and hypolimnion in the vertical water profile; as well as relevant moments of the reservoir’s hydrodynamic cycle, such as the well-established stratification (September 2018), the heterotrophic circulation (December 2018), the early stratification (April 2019), the late-stratification (September 2019) and the autotrophic circulation (January 2020). The definition of the heterotrophic and autotrophic circulations was done according to the metabolism that characterizes the water column at specific moments in time, and have been previously observed and characterized in the reservoir [28, 31]. Furthermore, during the studied period, the water level in the reservoir presented the low water level fluctuations that are observed under normal water availability years [22] (Fig. 1b).

The sampling was done in a representative central station of the reservoir [29, 30], where vertical profiles of Temperature (T), pH, and Dissolved Oxygen (DO) were obtained using an *In-Situ*® multiparameter sonde Aqua TROLL 500. Secchi depth data were taken using the method by Preisendorfer [32]. Water samples were collected throughout the water column (0, 1, 2, 4, 6, 8, 12, 16, 20, 24 m depths and at the lowest possible depth near the bottom) with an Uwitec sampling bottle, separating samples for biogeochemical characterization and microbial analyses as described below. Previous studies have shown that this central station is representative of the system [29, 33], and since September 2007, it has been used in the monitoring to ensure the long-term viability of our time series.

### Biogeochemical Characterization

Water samples for TN and TP determination were directly collected in polypropylene 50 mL bottles, while for dissolved inorganic species —NO_3_^−^, NO_2_^−^, NH_4_^+^, soluble reactive phosphorus (SRP) and soluble reactive silica (SRSi) —, 50 mL of water were filtered through a 0.22-µm Millipore® nitrocellulose membrane and preserved with 2 drops of chloroform. The samples were frozen at −20ºC for preservation until analysis. TP and TN samples were digested simultaneously as indicated by Valderrama [34]. Then both, totally digested and inorganic species samples, were analyzed by spectrophotometry using a Skalar San Plus segmented-flow auto-analyzer, using the standard methods by Grasshoff et al. [35] and the Kirkwood [36] protocol.

The content of Chlorophyll a (Chl-a) was determined in samples taken at 0, 1, 2, 4, 6 and 8,12, 16, 20 m depth and the deepest sample possible. Sixty milliliters of water were filtered through a 0.45 µm Millipore® nitrocellulose membranes, and conserved in 15-mL conical tubes covered with aluminum foil to protect them from light and stored at 4ºC in the dark until their analysis. Chl-a was extracted with 90% V/V acetone and the analytical determination was performed by spectrophotometry, and computed according to the Jeffrey and Humphrey [37] equations.

### 16S rRNA Gene Survey

For the characterization of the prokaryotic community (Bacteria and Archaea), a metabarcoding approach was used with a 16S rRNA gene survey. About 200–500 mL of water samples were filtered through a 0.22-µm membrane, and the membrane was stored at −4ºC until processing. Firstly, total DNA was extracted with the DNeasy PowerWater Kit (Qiagen) for the water samples. The 16S rRNA gene fragments were amplified with primers 515F/806R, targeting the bacterial and archaeal V4 region [38]. PCR reactions contained 2–20 ng of template DNA, 1X Takara ExTaq PCR buffer (plus Mg^2+^) (TaKaRa Corp., Shiga, Japan), 50 µmol L^−1^ Takara dNTP mix, forward and reverse primers (0.4 μmol L^−1^ final concentration), 0.625 U Takara Ex Taq DNA Polymerase and nuclease-free water up to 25 µL. The amplification protocol consisted of a denaturalization step at 95ºC (3 min), followed by 30 cycles of 95ºC (30 s), 50ºC (40 s), 72ºC (90 s), and a final extension at 72ºC (12 min).

The obtained amplicons contained the adapters for Illumina sequencing and a barcode sequence for identification. After amplifying in triplicates (3 times each in 25 µL PCR reaction), the desired amplicons were pooled and purified using the Agencourt AMPure XP PCR Purification system (Agencourt Bioscience Co.). Amplicons were paired-end sequenced on an Illumina MiSeq platform (Yale Center for Genome Analysis, CT, USA), where a total of 2,938,646 raw sequences were obtained (~ 250 bp) (Table S1).

### Sequence Analysis

The 16S rRNA gene sequence analysis was done with the QIIME2 package (https://qiime2.org) [39]. Briefly, the obtained paired-end sequences were demultiplexed and visualized for quality with the “q2-demux” plugin (https://github.com/qiime2/q2-demux). Sequences were merged and checked for quality with DADA2. DADA2 implements an algorithm that identifies sequences of exact bimeras (two-parent chimeras) using the Needleman-Wunsch global alignment [40]. The sequences resulted in 1,471,484 filtered non-chimeric sequences, with a minimum of 13,077 sequences per sample, suggesting enough sequences for this study purpose (Table S1 and Fig. S1). An abundance table was obtained with the representative sequences or Amplicon Sequence Variants (ASVs), meaning sequences with 100% nucleotide identity. These representative sequences were aligned with MAFFT [41] and a phylogenetic tree was generated with FastTree [42]. The taxonomy was assigned using the “q2-feature-classifier” plugin [43], and the Silva database as reference (silva-138–99–515–806-nb-classifier.qza) (v.138, Nov/2020). The chloroplast and mitochondrial sequences were removed from the 16S rRNA gene dataset, to maintain exclusively the sequences from Bacteria and Archaea (Figs. S2 and S3).

### Data and Statistical Analyses

To determine the trophic state of the reservoir, the Trophic State Index (TSI) of Carlson and the TSI of Kratzer and Brezonik were calculated for the sampled times using the equations proposed by Carlson [44], and Kratzer and Brezonik [45], respectively (Table S2). The thermal behavior of the reservoir, as well as the dissolved oxygen concentration were graphed in Surfer® (Golden Software, LLC) while the vertical profiles for each sampled time were graphed with GraphPad Prism 10.4.0 (GraphPad Software). To cluster the samples according to their physicochemical and biogeochemical characteristics and observe the main variables, a principal component analyses (PCA) was done with the “FactoMineR” and “factoextra” programs, using standardized data with the function “scale” [46].

Then, for the prokaryotic composition, the alpha and beta diversity analyses were done with the “phyloseq” program under an RStudio environment [47, 48]. The number of observed ASVs, as well as the Shannon and Simpson indexes, were calculated with the “estimate_richness” function in “phyloseq” excluding singletons [47]. The 16S rRNA gene relative abundance of the different prokaryotic groups were obtained also with “phyloseq” and “tydiverse” [49]. Graphics of the alpha diversity and the 16S rRNA gene relative abundance graphics were done with GraphPad Prism 8.4.2 (GraphPad Software).

The beta analyses were done with a Principal Coordinate Analysis-Unifrac (PCoA) using the “ordinate” function, also in “phyloseq” [50, 51]. The differences in the communities among compartments were verified with the function “betadisper” in “vegan,” followed by a PERMANOVA test (“ANOVA” and “permutest”). The relationship between the 16S rRNA gene assemblage structure and the physicochemical and biogeochemical characteristics was calculated with a canonical correspondence analysis (CCA) using the function “cca” in ‘vegan’ [52]. The physicochemical data were firstly transformed using the “preprocess” function in “caret,” with the *center* and *scale* options [53], while the 16S rRNA gene data was used in a relative abundance format. The physicochemical parameters with *p*-values < 0.05 were selected with “envfit” function, also in “vegan.” All these analyses were done program under an RStudio environment [48].

## Results

### Physicochemical and Biogeochemical Characterization

**Fig. 2:**
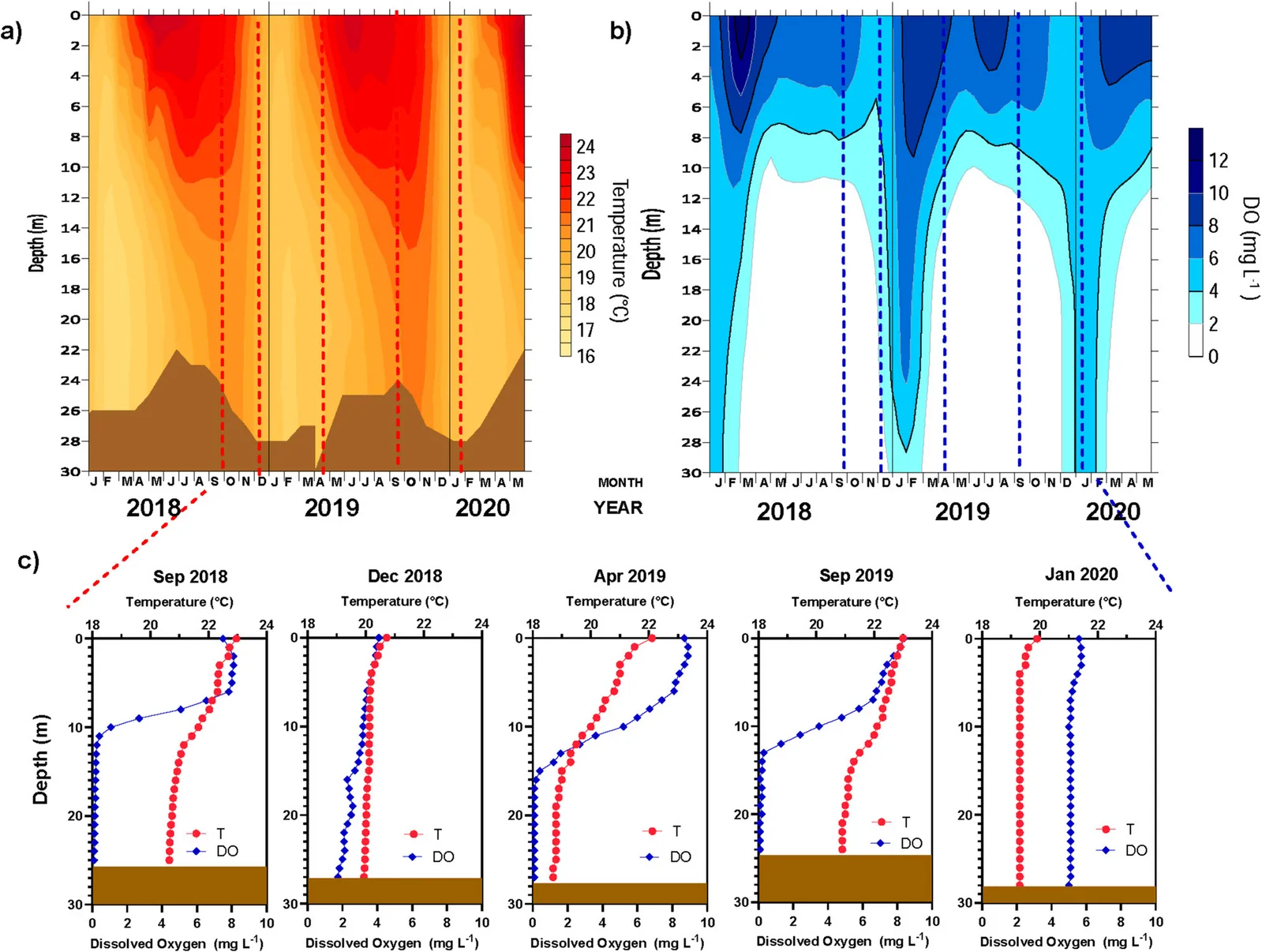
Spatio-temporal distribution of Temperature and Dissolved Oxygen (DO) in Valle de Bravo Reservoir from September 2018 to January 2020. **a**) Contour maps of the evolution of Temperature (yellow to red gradient) and Dissolved Oxygen (DO) (white to blue gradient) over time, where months are abbreviated by its initial letter (v.g. J, January; F, February; etc.). **b**) Vertical profiles of Temperature (°C) and DO (mg L^−1^) measured during the 5 samplings (September 2018, December 2018, April 2019, September 2019 and January 2020). The brown box represents the sediment layer

**Fig. 3:**
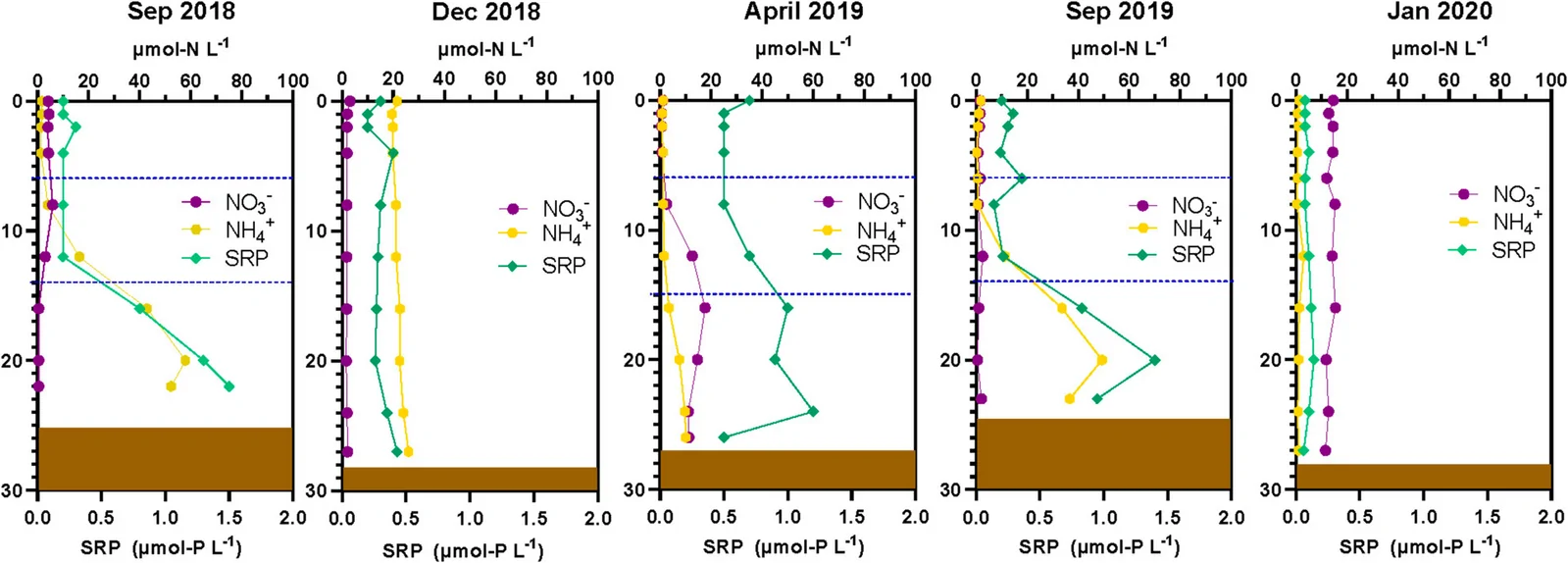
Evolution of nutrient’s vertical profiles in the Valle de Bravo Reservoir. Vertical distribution of nitrate (NO_3_^−^, in purple), ammonia (NH_4_^+^, in yellow) (scale for both on the top axis), and Soluble Reactive Phosphorous (SRP, green) on the bottom axis, along the samplings (September 2018, December 2018, April 2019, September 2019 and January 2020). The brown box represents the sediment layer, while the dashed blue lines indicate the beginning and the end of the oxycline

During the studied period of L-WLF, VB presented eutrophic conditions (Table S2) and behaved as a warm monomictic water body with an annual circulation during winter (October- January), while the rest of the year remained thermally stratified (Fig. 2a); which was reflected in the DO content of the water column along the year (Fig. 2b and Table S3). The sampled months represented the most relevant periods of the cycle: i) well-established stratification (Sep.2018) characterized by thermal and DO stratification, with an epilimnion (0–6 m depth) where DO was oversaturated (> 7.02 mg L^−1^ = 100% DO saturation for VB) (Fig. 2c), and where DIN and SRP were almost depleted (Fig. 3), while in the hypolimnion (from 17 m depth to the bottom) NH_4_^+^ and SRP accumulated and DO was depleted. ii) Heterotrophic circulation (Dec.2018) characterized by sub-oxic conditions (30–58% of DO saturation), the presence of SRP (0.28 µmol-P L^−1^) and NH_4_^+^ (21.3 µmol-N L^−1^) in the whole water column, and the depletion of NO_3_^−^ (0.52 µmol-N L^−1^). iii) Early stratification (Apr.2019), with a high temperature gradient between epilimnion and hypolimnion and a DO gradient. NO_3_^−^ became the dominant DIN species in the metalimnion and hypolimnion, reaching the highest values at 12–20 m, while NH_4_^+^ was almost depleted in the most of water column with exception of the bottom. iv) Late stratification (Sep.2019) characterized by the presence of thermal and DO gradients, with an anoxic hypolimnion rich in NH_4_^+^ and SRP, with NO_3_^−^ almost depleted. And finally, v) the autotrophic circulation (Jan.2020) characterized by a slight sub-oxygenation (73–78% of DO saturation) of the water column, a dominance of NO_3_^−^ (13.77 µmol-N L^−1^) among the DIN species and the depletion of NH_4_^+^ (1.02 µmol-N L^−1^) (Figs. 2 and 3).

**Fig. 4:**
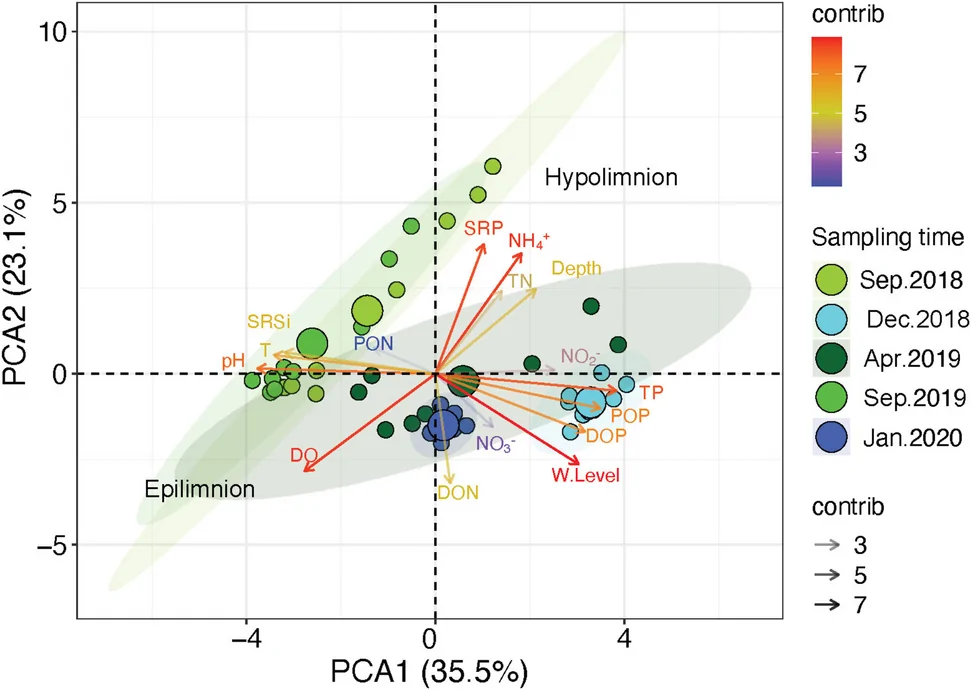
Principal component analysis (PCA) of the physicochemical and biogeochemical parameters, along the 5 sampled months (September 2018, December 2018, April 2019, September 2019, and January 2020). The horizontal and vertical axes of the figure correspond to PCA1 and PCA2, respectively. The shaded ellipses represent the 5 defined groups corresponding to each sampling month. The size of each ellipse reflects the variance of the data within each group. Green circles and shades describe the stratification months (September 2018 and September 2019), while cyan circles and shade corresponds to the heterotrophic circulation month (December 2019) and the dark-blue circle and shade to the autotrophic circulation month (January 2020)

The PCA analysis clustered the samples mainly according to the sampling time. The stratification periods were defined by a higher dispersion of the samples within the clusters, separating those of the epilimnion and the hypolimnion (Fig. 4). The epilimnion samples were characterized by higher pH, T, SRSi, and DO values, while the hypolimnion was associated to the increase of depth, and of SRP, PT and NH_4_^+^ concentrations. The early stratification period (April.2019) was located between the well-established and late stratifications, and the circulations periods. Samples from both circulations were closely graphed; the heterotrophic circulation (Dec.2018) was associated with higher concentrations of TP and organic P-species (DOP and POP), while the autotrophic circulation (Jan.2020) was characterized by high concentrations of NO_3_^−^ and DON.

### The Prokaryotic Community

**Fig. 5:**
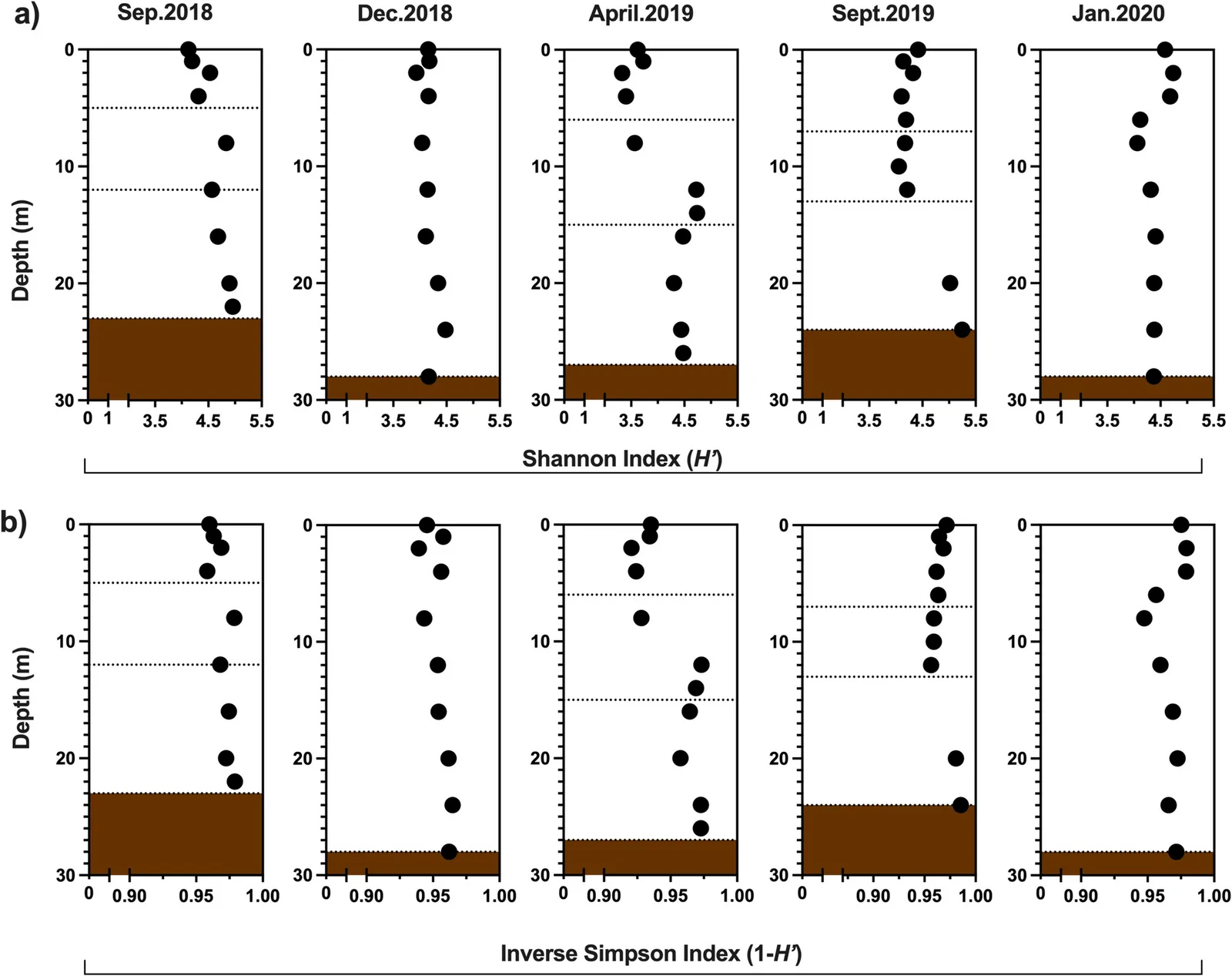
Alpha diversity indicators derived from 16S rRNA gene libraries in the water column from Valle de Bravo reservoir. Samples were collected at the central monitoring station at different depths, during September 2018, December 2018, April 2019, September 2019 and January 2020. **a**) Shannon diversity index (*H’*), and **b**) Inverse Simpson diversity index (1-*D*’)

The prokaryotic community was studied by surveying the 16S rRNA genes associated with Bacteria and Archaea. The diversity indicators, such as the Shannon index (*H*’) and the inverse Simpson (1-*D*’) indexes, showed similar values along the water column during the circulation periods (Dec.2018 and Jan.2020); yet values tended to increase with depth during the stratifications (Aug.2018, Apr.2019 and Sep.2019), with the largest values at the bottom (Fig. 5). The early stratification (Apr.2019) showed the highest difference between the average Shannon diversity index of the epilimnion and the metalimnion (∆0.82), followed by the well-established stratification (∆0.41), while in the late stratification this value was similar between both layers, being slightly higher in the epilimnion (∆−0.08). The hypolimnion showed the highest diversity in the late stratification (5.0–5.2). For the circulations, the *H*’-values were similar along the water profile, with an average value of 4.17 for Dec.2018, and slightly higher in the autotropic circulation (4.40), being both values significantly different (*p*-value = 0.0119). A similar significant difference was found in the inverse Simpson value for the circulation periods (*p*-value = 0.0045), and with the highest value in the autotrophic circulation (0.9676) than in the heterotrophic one (0.9539).

**Fig. 6:**
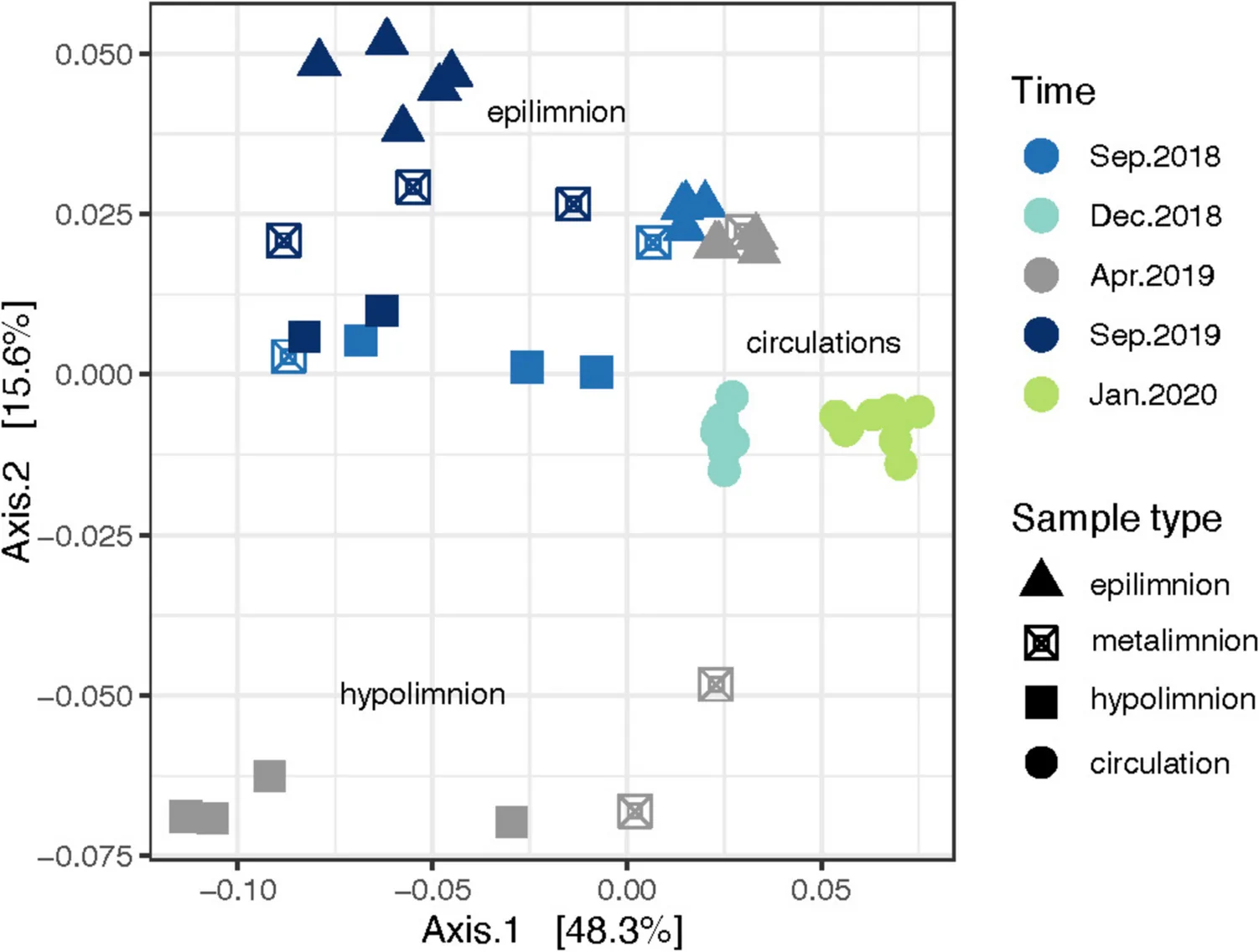
Principal Coordinate Analysis-Unifrac (PCoA) derived from 16S rRNA gene libraries in the water column of Valle de Bravo reservoir. Samples were collected at the central monitoring station, during September 2018, December 2018, April 2019, September 2019 and January 2020 (sampling time). Figure shapes depend on the sample type: epilimnion, metalimnion, hypolimnion and circulation

Regarding the structure of the prokaryotic assemblage, the PCoA analysis showed that the samples were clustered by their position in the water column and/or the hydrodinamic period (Fig. 6). For example, the samples from the circulation periods were clustered by the circulation type, heterotrophic (Dec. 2018) and autotrophic (Jan.2020), being both significantly different compared to the stratifications, and between them (*p*-value 0.004). The samples of the epilimnion were similar for the early and well-established stratifications, while those from the late stratification (Sep.2019) were different. In contrast, the hypolimnion samples from the well-established and late stratifications were similar, but different from those of the early stratification (Apr.2019).

**Fig. 7:**
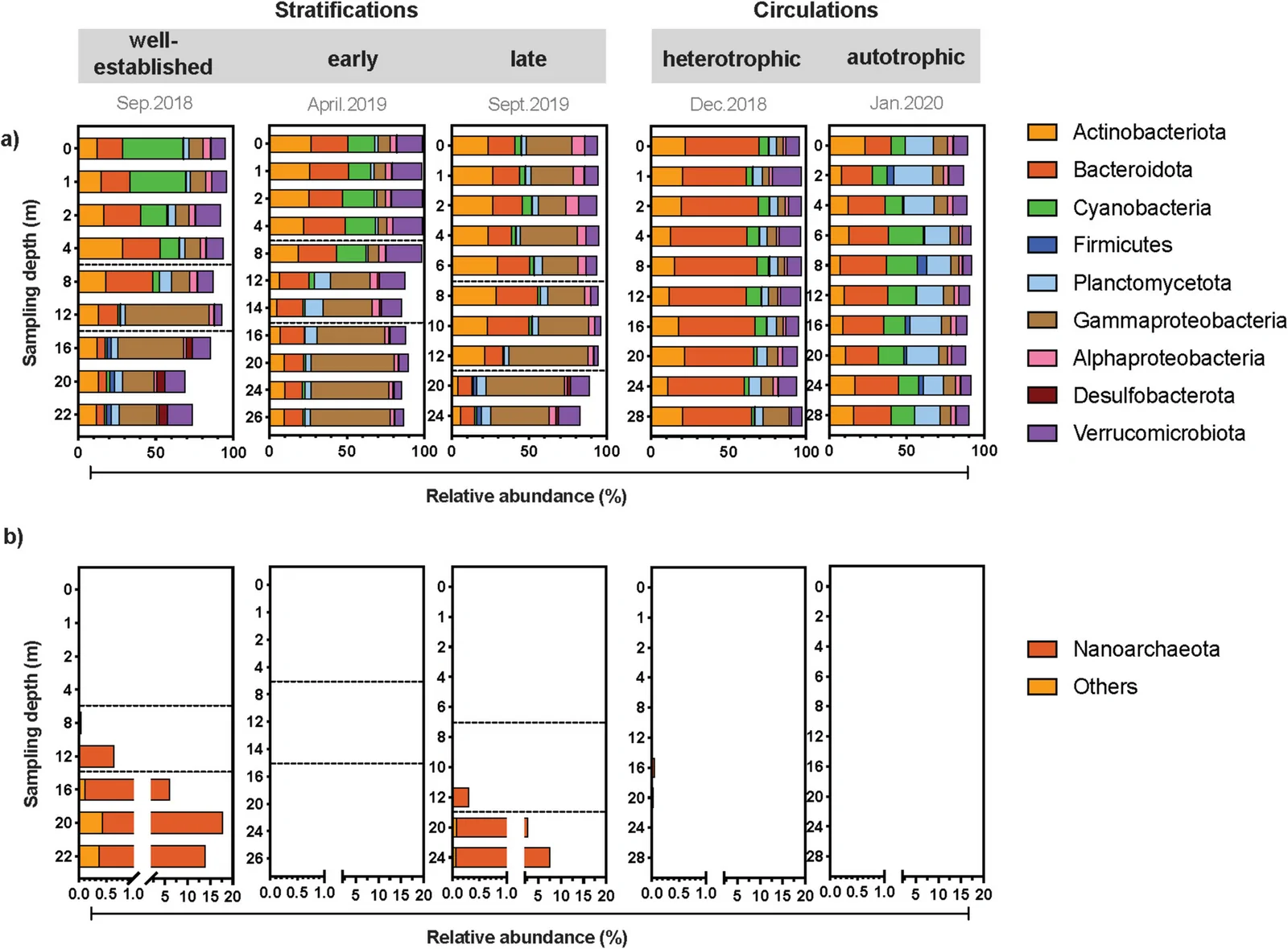
Relative 16S rRNA gene abundance of the main phyla found in the central monitoring station of the Valle de Bravo reservoir, separated by the studied stratifications and circulation periods (September 2018, December 2018, April 2019, September 2019 and January 2020), **a**) bacteria and **b**) Archaea

The abundance and taxonomic affiliation of the diverse prokaryotes was also in accordance with the sampling depth and time. The main groups included *Actinobacteriota, Bacteroidota, Cyanobacteria, Firmicutes, Planctomycetota, Gammaproteobacteria, Alphaproteobacteria, Desulfobacterota,* and *Verrucomicrobiota* (Fig. 7a), where their abundance showed some patterns. For example, Cyanobacteria mas mainly found in the epilimnion and in the circulation, being most abundant during the autotrophic circulation (8–22%) and in the early stratification (14–21%). *Bacteroidota* was the main phyla during the heterotrophic circulation, accounting for 41 to 50% of the relative abundance, followed by *Actinobacteriota* (11–22%) and *Verrucomicrobiota* (7–19%). In the autotrophic circulation, *Planctomycetota* accounted for 13–20% of the 16S rRNA genes besides *Cyanobacteria*. At both circulation processes, *Gammaproteobacteria* and *Alphaproteobacteria* were at the lowest relative abundance compared to other periods; yet these classes accounted for 5.5–60% and 0.63–8.17%, respectively during the stratifications. In this sense, *Gammaproteobacteria* was the most abundant during the late stratification (17–51%), together with *Actinobacteriota* (4.3–30%) and *Bacteriodota* (9–27%). *Desulfobacterota* was also detected in the hypolimnion, mainly during the well-established stratification (4.0–5.8%).

Microorganisms belonging to *Archaea* were also found in this 16S rRNA gene survey of the reservoir. *Archaea* was mostly relevant in the hypolimnion for the well-stablished and late stratification, reaching up to 17.4% of relative abundance, with members mainly belonging to *Nanoarchaeota* (Fig. 7b). These archaeal sequences were detected in the hypolimnion and at the bottom of the metalimnion, being most abundant in the well-stablished stratification, and were mostly affiliated to the order *Woesearchaeales* (Fig. S4a). Sequences from other phyla were also detected but they contributed at the most with a 0.44% of the relative abundance of the 16S rRNA genes (well-established stratification). These other phyla included members of *Micrarchaeota*, *Iainarchaeota*, *Halobacterota*, *Euryarchaeota*, *Crenarchaeota*, and *Thermoplasmatota*; yet most of these sequences were related to methanogens (Fig. S4b).

The Canonical Correspondence Analysis (CCA) showed that all the physicochemical and biogeochemical parameters measured were significantly correlated with the structure of the prokaryotic community from the water column (Table 1, Fig. S5), where SRP and DO were the strongest factors. An insight into the stratifications showed that all the parameters had a significant correlation with the community structure, except for NO_2_^−^ (Fig. S6). In this case, there was a positive relationship between prokaryotes from the hypolimnion in the well-established and late stratifications and the SRP and NH_4_^+^, while a correlation with TP was observed for the samples from the hypolimnion in the early stratification.

**Table 1:** CCA analyses results, showing the values of adjusted canonical coefficients (*r*2) and level of significance (*p*-value), for the physicochemical characteristics in the prokaryotic community of the Valle de Bravo reservoir

*Compartment* Parameter	*r*^*2*^	*Pr*(> *r*)	Significance
*Water column*			
Depth	0.416	0.001	***
Temperature	0.5826	0.001	***
DO	0.6987	0.001	***
pH	0.2009	0.005	**
SRSi	0.2553	0.003	**
NH_4_^+^-N	0.6291	0.001	***
NO_2_^−^-N	0.6118	0.001	***
NO_3_^−^-N	0.4134	0.001	***
DIN	0.6558	0.001	***
TN	0.2901	0.001	***
SRP	0.7669	0.001	***
TP	0.2352	0.005	**

^†^ Significance codes:. *p*-value < 0.10, *p-value < 0.05, **p-value < 0.01, ***p-value < 0.001

## Discussion

The diversity, structure, and composition of the prokaryotic community in VB is highly correlated with the biogeochemical parameters along the spatio-temporal variations associated to the hydrodynamic behavior of the reservoir during a L-WLF period. The water level varied smoothly during this period with lower levels in September (Sep.2018 and Sep.2019), and higher levels during winter (Dec.2018 and Jan.2020), when the reservoir reached 95% of its higher water volume capacity (a maximum of 391 × 10^6^ m^3^) (Dec.2018, Jan.2020), where the reservoir reached 95% of its water volume capacity (maximum 391 × 10^6^ m^3^) (Fig. S7) [29, 54]. During this L-WLF period, VB behaved as a warm monomictic water body as reported previously by Merino-Ibarra et al. [29]. This hydrodynamic cycle encompasses the diversity and composition prokaryotic community during the circulation and stratification distributed along the water column, as observed in other water bodies [55–58]. In this way, the distribution and composition of the microbial communities primarily exhibits temporal variations during the circulation period, whereas spatial variations where mainly along the water column and found during the stratification of the different water layers.

### The Prokaryotic Community During Circulation

Circulation periods are known to homogenize the physicochemical conditions along the water column in monomict systems [20]. The two circulation samplings here studied were of distinct nature. At the beginning of the winter (here as December 2018), a heterotrophic circulation occurs. At this moment, the breakdown of thermal gradient leads to a homogeneous distribution of the biogeochemical parameters, specifically DO, SPR, and NH_4_^+^, along the water column. The presence of DO and nutrients promotes the dominance of aerobic and facultative heterotrophic bacteria such as those from *Bacteroidota, Actinobacteriota,* and *Verrucomicrobiota*, which, in this case, accounted for 73 to 81% of the relative abundance of the 16S rRNA genes in the water column. These phyla have been often reported in inland water bodies [59, 60]. At this time, *Flavobacterium* (*Bacteroidota*) was one of the most dominant genera in the samples. Blooms of *Flavobacteria* populations have been reported in eutrophic lakes associated with resource availability, shaping heterotrophic seasonal succession [61]. Bacteria from the hgcI_clade (*Actinobacteriota*, *Sporichthyaceae*), *Fluvicola* (*Bacteroidota*), and *Terrimicrobium (Verrucomicrobiota*) were the most abundant, which have been also observed in eutrophic lakes associated with winter samples [60].

Autotrophic circulation (here in January 2020) follows heterotrophic circulation, and it is characterized by an increase of DO concentrations and the shift of the DIN dominant specie from NH_4_^+^ to NO_3_^−^. In this period, the abundance of *Bacteroidota* decreased, representing 24% of relative abundance, while *Cyanobacteria* increased up to 23%. This might be driven by the availability of SRP and DIN after the onset of circulation, promoting the autotrophic prokaryotic community [23, 28, 31, 55]. This shift in the metabolism of the oxygenic photosynthetic communities may explain the increase in the amount of DO, and, in some extend, the decrease of NH_4_^+^. These observations align with the previous metabolic studies performed in the reservoir [28, 31] which reported that respiration was the dominant process in the early circulation in December, whereas the production of organic matter surpassed respiration during late circulation, in January.

### The Prokaryotic Community During the Stratification

During the stratification months, the main variation in the physicochemical and biogeochemical distributions was an intensification of the gradients between the epilimnion and the hypolimnion. As expected, the epilimnion temperature was slightly higher during the early stratification of April 2019 than for in the two stratifications sampled. This occurs because in the central highlands of Mexico the highest temperature is reached just before the onset of the rainy season (June–October), when temperature drops slightly [29]. Because stratification was establishing the bottom of the oxycline appeared deeper than the other two stratifications, and the coexistence of SRP, NH_4_^+^ and NO_3_^−^ species in the hypolimnion also occurred. This was noticed in the prokaryotic diversity indicators, where the ∆*H*’ value between the epilimnion and metalimnion suggests the shift from a low-diversity period (winter circulation) to a stratified water column (Apr.2019) that promotes the diversification of environments and metabolisms, and therefore, of the prokaryotic diversity commonly found in stratified water columns [62].

During the following stratification months, the vertical segregations of the physicochemical and biogeochemical parameters were also reflected in the alpha diversity indicators. The values of the Shannon index slightly increased with depth during the stratification, possibly corresponding with the presence of different biogeochemical anaerobic pathways, as reported in other tropical aquatic systems [58, 62–65]. Yet, despite the similar physicochemical and biogeochemical characteristics of the epilimnion across stratifications, there were notable differences in the composition of the prokaryotic communities. For example, *Cyanobacteria* was the dominant phyla in the epilimnetic microbial community during the well-established stratification. However, during the late stratification of September 2019, the epilimnion transitioned to being dominated by heterotrophic phyla (*Bacteroidota* and *Actinobacteria*), while *Cyanobacteria* had a maximum of 6% of relative abundance. These findings align with the previous studies of the phytoplanktonic composition [23] and metabolism [28] and could be related to the effect of boundary mixing events form the hypolimnion reported by Merino-Ibarra et al. [30].

**Fig. 8:**
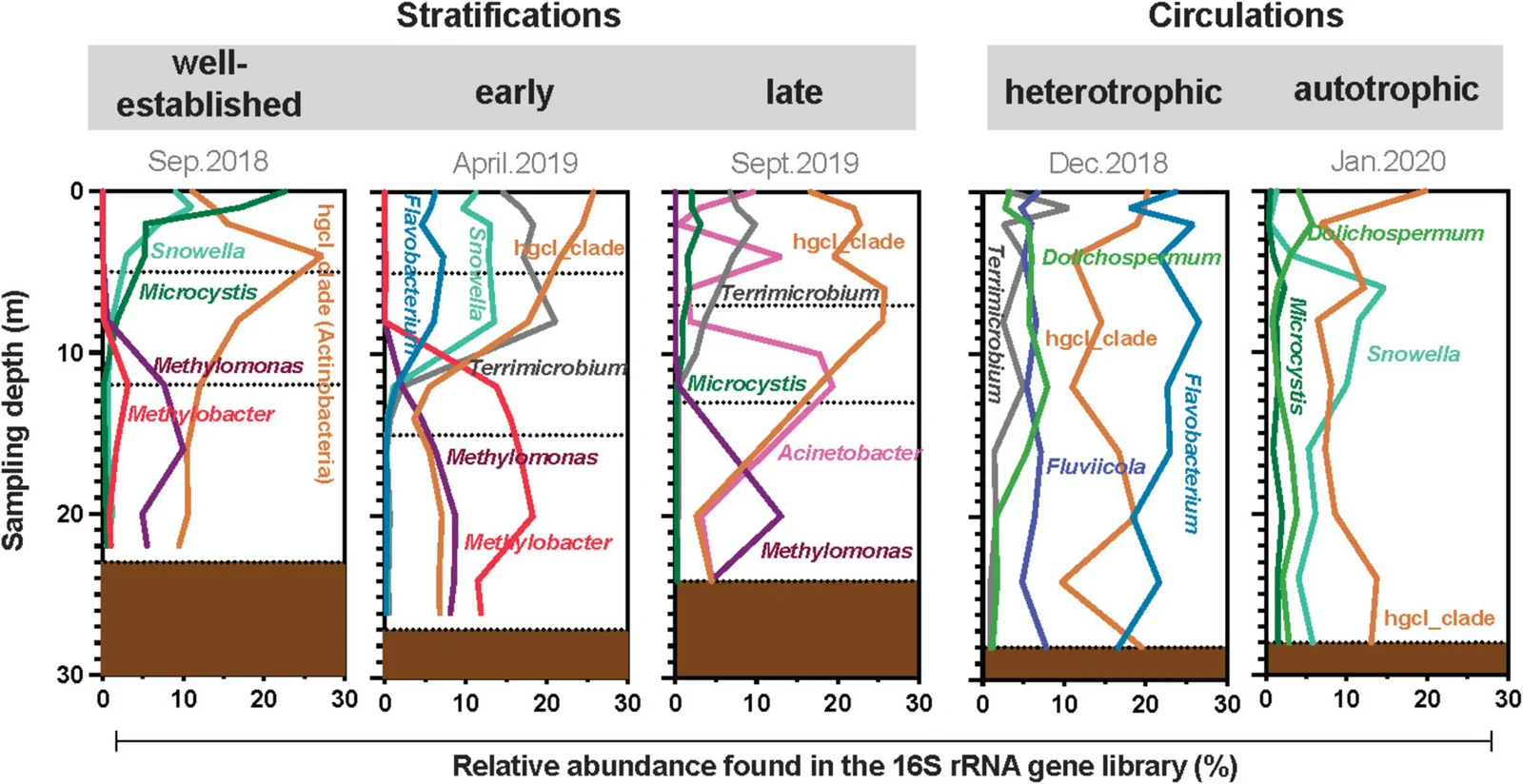
Relative 16S rRNA gene abundance of the main genera found in the central monitoring station of the Valle de Bravo reservoir, separated by the studied stratifications and circulation periods (September 2018, December 2018, April 2019, September 2019 and January 2020)

In the hypolimnion, the prokaryotic diversity was higher than in the epilimnetic layer, and the composition and structure varied according to the duration of stratification (Figs. 5 and 6). Some heterotrophic phyla such as *Actinobacteria* was detected in this water layer; yet a remarkable feature is the presence of methanotrophs such as *Methylomonas* and *Methylobacter* (both *Methylomonadaceae*) in a relative abundance of up to 13 and 18%, respectively, in the early stratification (Fig. 8). Genera such as *Methylobacter*, *Methylomonas* and *Methylomicrobium* are dominant and often found in the water column of stratified lakes [66–70]. Its activity occurs when CH_4_ diffuses from deep anoxic water layers or sediments, and can be oxidized at the bottom of the oxycline; however, this oxidizing activity has been registered in oxygen deficient conditions [71, 72]. Aerobic methanotrophs such as those belonging to *Methylomonadaceae* and *Methylocystaceae* have been reported as been more versatile than previously thought and can be involved in the anaerobic oxidation of methane using nitrate/nitrite or some metals under oxygen limitation [73].

At the well-established stratification, sulfate-reducers (*Desulfobacterota*) were found in the hypolimnion (up to 6%), while its presence diminished by the late stratification (up to 2.3%). This redox shift in the bottom of the water column was also visualized by the presence of methanogens such as *Methanomicrobiales* and *Methanobacteriales* in the water column with nearly 0.3% (Fig. S4b). The abundance of these methanogens in the well-established stratification matches with maximum CH_4_ emission register in the reservoir during the same period [54]. However, it must be considered that the use of relative abundances based on the 16S rRNA gene libraries is a preliminary survey of the potential impact that some prokaryotes might have in the biochemical processes. Future studies must then consider quantitative approaches such as quantitative PCR (qPCR) of functional genes, as well as measuring the production or consumption of metabolites to better explain the potential function of microbial communities.

Finally, the importance of nitrifiers have been outlined among the microbial processes in aquatic systems [8] and potential nitrifiers were also found in the water column of VB. For the well-established stratification, the abundance of bacteria belonging to *Nitrosomonadaceae* was noticed up to 1.8% from 12 to 26 m depth (Fig. S9), where the simultaneous presence of NO_3_^−^ and NH_4_^+^ (Fig. 3), as well as NO_2_^−^ (Fig. S10). In this way, NH_4_^+^ can be oxidized to NO_2_^−^ by *Nitrosomonadaceae*, and then NO_2_^−^ oxidized to NO_3_^−^ by *Nitrospiraceae*. *Nitrospira* is an often-found nitrite oxidizer, as well as grow in oxygen fluctuating environments [74]. For the well-established stratification (Sep.2018), *Nitrosomonadaceae* was mainly found in the epilimnion, with a maximum of 1.6% of relative abundance in the surface and at 1 m depth, which also matches with the presence of NO_2_^−^ mainly in this water layer (Fig. S10). During the autotrophic circulation, the three N-species were detected along the water column, as well as some nitrifiers (up to 0.5% of relative abundance).

Despite the inherent limitations of metabarcoding techniques for exploring the archaeal community with the primers here used [75], our results describe for the first time the archaeal community of the reservoir of Valle de Bravo, which was relevant during the well-established and late stratification in the hypolimnion and the bottom of the metalimnion (Fig. 7b). This archaeal composition matches with the taxonomical distribution of archaea found in freshwater lakes. A study including data from 109 lakes showed that *Woesearchaeia*, *Methanomicrobia* and *Nitrosophaeria* were the main archaeal contributors [76], were *Woesearchaeia* was dominant in the dataset. It has been estimated that the commonly used primers 515F/806r [38] produce only 2.1% of the archaeal sequences on average [75], so future studies in VB must include archaeal-specific sequencing surveys to reveal the diversity and representatives of these *Woesearchaeota*, as well as other archaeal groups, to better understand the role of this relevant group of microorganisms.

Our results also showed the presence of important prokaryotes related to processes such as autotrophy, heterotrophy, methanotrophy, sulfate reduction, methanogenesis, and nitrification. However, complementary biogeochemical determinations must be done to confirm and estimate the activity of these microorganisms. Furthermore, future spatial microbiological surveys are also in the prospective continuity of the monitoring to address potential differences between deep and shallow areas in the reservoir, including near the river entrances, and pump-up and water withdrawal spots, giving us a better insight into the distribution of microorganisms in this valuable system.

Our findings imply that the management strategies must consider the succession of the prokaryotic community in dependence of circulation-stratification hydrodynamic cycle in L-WLF periods. For example, focusing on avoiding the high abundance of Cyanobacteria during the stratification and heterotrophic circulation by preventing high loads of nutrients (P and N) by water injections, which has been associated to the detriment of water quality and potential toxicity of aquatic systems [77, 78], including VB [79, 80].

## Conclusions

This study provides an insight into the prokaryotic community of a high-relevance tropical eutrophic reservoir, considering a low-water level fluctuation period near its maximum capacity, which is a key time that helps to establish baseline conditions for future studies. The spatio-temporal distribution, composition, and diversity of the prokaryotic community were closely linked to the physicochemical and biogeochemical parameters, driven by the circulation-stratification hydrodynamic cycle of the reservoir. The epilimnion samples were characterized by higher pH, T, SRSi, and DO values where *Cyanobacteria* were abundant. While the hypolimnion was associated to the increase of SRP, PT, and NH_4_^+^ concentrations, as well as the increase of the prokaryotic diversity, possibly associated with the diversity of metabolisms under anaerobic conditions. This metabarcoding approach complements previous research in the site about the ecosystemic metabolism of C, N, and P, and taxonomic studies of phytoplankton, providing a platform for future studies to better understand the biogeochemical processes in the reservoir.

## Supplementary Information

Below is the link to the electronic supplementary material.

Supplementary file1 (DOCX 1205 KB)

## Data Availability

The sequence data is in a publicly accessible repository, available in the NCBI BioSample database (http://www.ncbi.nlm.nih.gov/biosample/) under accession numbers SAMN45085681 (Sep.2018), SAMN45085682 (Dec.2018), SAMN45085683 (Apr.2019), SAMN45085684 (Sep.2019), SAMN45085685 (Jan.2020) (BioProject PRJNA1191546).
