# Supplementary material for: Diversity and Structure of the Prokaryotic Community in Tropical Monomictic Reservoir
Source: Microb Ecol. 2025 Mar 12;88(1):12. doi: 10.1007/s00248-025-02508-1 (PMC11903632; doi:10.1007/s00248-025-02508-1)
Supplement: Supplementary file 1 — Supplementary file1 (DOCX 1205 KB) [file 248_2025_2508_MOESM1_ESM.docx]

**Diversity and structure of the prokaryotic community in tropical monomictic reservoir**

Mariel Barjau-Aguilar^1^, Ana MJ Reyes-Hernández^2^, Martín Merino-Ibarra^3^, Gloria Vilaclara^4^, Jorge A Ramírez-Zierold^3^, Rocío J Alcántara-Hernández^1^*.

^1^Instituto de Geología, Universidad Nacional Autónoma de México, Ciudad Universitaria, Av. Universidad 3000, Del. Coyoacán, 04510, Ciudad de México, México.

^2^Posgrado en Ciencias Biológicas, Universidad Nacional Autónoma de México, Unidad de Posgrado, Edificio D, 1° Piso, Circuito de Posgrados, Ciudad Universitaria, Coyoacán, 04510, Ciudad de México, México.

^3^Unidad Académica de Biodiversidad Acuática, Instituto de Ciencias del Mar y Limnología, Universidad Nacional Autónoma de México; Av. Universidad 3000, Ciudad Universitaria Coyoacán, C.P. 04510, Ciudad de México, México.

^4^Grupo de Investigación en Limnología Tropical, FES Iztacala, Universidad Nacional Autónoma de México, Tlalnepantla, 54090, Estado de México, México.

Running head: Prokaryotes in a tropical eutrophic reservoir

*Corresponding author: Rocío J Alcántara-Hernández. Instituto de Geología, Universidad Nacional Autónoma de México, Ciudad Universitaria, Av. Universidad 3000, Del. Coyoacán, 04510, Ciudad de México, México, E mail: ralcantarah@geologia.unam.mx

| Table S1 Number of raw, filtered, and non-chimeric sequences obtained in this study, and the amplicon sequence variants (ASVs) found. | | | | |
| --- | --- | --- | --- | --- |
| Sample | Raw sequences | Filtered | Non-chimeric | ASVs |
| Sep.2018 | | | | |
| 2018.09.Z00 | 53945 | 30251 | 25273 | 269 |
| 2018.09.Z01 | 118685 | 71301 | 60020 | 380 |
| 2018.09.Z02 | 115227 | 69177 | 60268 | 438 |
| 2018.09.Z04 | 75038 | 43067 | 36120 | 335 |
| 2018.09.Z08 | 68806 | 38874 | 34237 | 396 |
| 2018.09.Z12 | 78274 | 41609 | 31107 | 394 |
| 2018.09.Z16 | 83237 | 45263 | 36099 | 428 |
| 2018.09.Z20 | 71149 | 41471 | 34731 | 525 |
| 2018.09.Z22 | 58226 | 33919 | 28678 | 481 |
| Dic.2018 | | | | |
| 2018.12.Z00 | 59085 | 36105 | 31318 | 287 |
| 2018.12.Z01 | 40839 | 24171 | 22078 | 263 |
| 2018.12.Z02 | 29928 | 17601 | 15606 | 200 |
| 2018.12.Z04 | 31180 | 17081 | 15158 | 203 |
| 2018.12.Z08 | 40033 | 21916 | 19536 | 220 |
| 2018.12.Z12 | 35541 | 19338 | 17459 | 208 |
| 2018.12.Z16 | 27561 | 14461 | 13077 | 203 |
| 2018.12.Z20 | 35343 | 19714 | 17799 | 249 |
| 2018.12.Z24 | 42243 | 23135 | 21081 | 268 |
| 2018.12.Z28 | 32983 | 19107 | 17233 | 238 |
| Apr.2019 | | | | |
| 2019.04.Z00 | 41386 | 24273 | 21500 | 187 |
| 2019.04.Z01 | 102707 | 60104 | 53132 | 270 |
| 2019.04.Z02 | 31195 | 17804 | 15872 | 155 |
| 2019.04.Z04 | 28524 | 16911 | 15276 | 157 |
| 2019.04.Z08 | 53908 | 31972 | 27978 | 211 |
| 2019.04.Z12 | 40384 | 23840 | 21042 | 314 |
| 2019.04.Z14 | 52458 | 28967 | 26769 | 383 |
| 2019.04.Z16 | 40785 | 22777 | 19981 | 292 |
| 2019.04.Z20 | 44443 | 24854 | 20858 | 274 |
| 2019.04.Z24 | 43858 | 24411 | 21023 | 287 |
| 2019.04.Z26 | 49307 | 26232 | 22629 | 306 |
| Sep.2019 | | | | |
| 2019.09.Z00 | 142607 | 84053 | 73881 | 391 |
| 2019.09.Z01 | 54221 | 26344 | 23048 | 225 |
| 2019.09.Z02 | 56577 | 31911 | 29158 | 280 |
| 2019.09.Z04 | 53527 | 31859 | 28096 | 258 |
| 2019.09.Z06 | 57186 | 31819 | 28008 | 243 |
| Sample | Raw sequences | Filtered | Non-chimeric | ASVs |
| 2019.09.Z08 | 65931 | 36092 | 32573 | 274 |
| 2019.09.Z10 | 57071 | 33277 | 28766 | 223 |
| 2019.09.Z12 | 61723 | 34616 | 28988 | 307 |
| 2019.09.Z20 | 59752 | 32360 | 27551 | 485 |
| 2019.09.Z24 | 94838 | 53534 | 46538 | 646 |
| Jan.2020 | | | | |
| 2020.01.Z00 | 62184 | 37295 | 33076 | 313 |
| 2020.01.Z02 | 108201 | 62691 | 59958 | 433 |
| 2020.01.Z04 | 55590 | 33892 | 30611 | 321 |
| 2020.01.Z06 | 57184 | 33125 | 29818 | 285 |
| 2020.01.Z08 | 75125 | 44618 | 40104 | 308 |
| 2020.01.Z12 | 75378 | 44942 | 40390 | 340 |
| 2020.01.Z16 | 51708 | 28120 | 25658 | 281 |
| 2020.01.Z20 | 47769 | 26725 | 24204 | 272 |
| 2020.01.Z24 | 46279 | 25954 | 22972 | 266 |
| 2020.01.Z28 | 29517 | 17040 | 15148 | 232 |
| Minimum | **27,561** | **14,461** | **13,077** |  |
| Maximum | **142,607** | **84,053** | **73,881** |  |
| TOTAL | 2,938,646 | 1,679,973 | 1,471,484 |  |

**Fig. S1** Rarefaction curve of the samples analysed for this study.

**Fig. S2** Relative abundance of chloroplasts sequences eliminated from the 16S rRNA gene data base for this study.

**Fig. S3** Relative abundance of mitochondrial sequences eliminated from the 16S rRNA gene data base for this study

| **Table S2** Trophic State Indexes (TSI) –Carlson and Kratzer and Brezonik– and external inputs of TP and TN for the sampled months of Valle de Bravo reservoir. | | | | | | | | | | | | | | |
| --- | --- | --- | --- | --- | --- | --- | --- | --- | --- | --- | --- | --- | --- | --- |
| **Date** | **Secchi** | **Chl-a** | **TP** | **TN** | **TSI (SD)** | **TSI (CHL)** | **TSI**  **(TP)** | **TSI Carlson** | | **TSI**  **(TN)** | **TSI**  **Kratzer and Brezonik** | | **TP**  **input** | **TN**  **input** |
|  | **(m)** | **(mg L^-1^)** | **(mg P L^-1^)** | **(mg N L^-1^)** |  |  |  |  |  |  |  |  | **(mg P m^2^ d^-1^)** | **(mg N m^2^ d^-1^)** |
| **2018.09** | 1.28 | 264.14 | 39.55 | 830.12 | 56.5 | 85.3 | 57.2 | 66.3 | Eutrophic | 151.4 | 87.6 | Eutrophic | 5.23 | 79.80 |
| **2018.12** | 3.60 | 36.80 | 141.52 | 813.26 | 41.5 | 66.0 | 75.6 | 61.0 | Eutrophic | 151.1 | 83.6 | Eutrophic | 5.61 | 29.99 |
| **2019.04** | 1.3 | 177.88 | 89.61 | 913.92 | 56.2 | 81.4 | 69.0 | 68.9 | Eutrophic | 152.8 | 89.9 | Eutrophic | 3.32 | 25.59 |
| **2019.09** | 0.85 | 197.86 | 31.52 | 754.88 | 62.3 | 82.5 | 53.9 | 66.2 | Eutrophic | 150.1 | 87.2 | Eutrophic | 5.33 | 63.35 |
| **2020.01** | 2.83 | 111.59 | 51.41 | 746.20 | 45.0 | 76.9 | 61.0 | 60.9 | Eutrophic | 149.9 | 83.2 | Eutrophic | 2.77 | 57.67 |

Chl-a, TP and TN are average values obtained for the whole water column profile. Equations used: TSI (*SD*)=60-14.41 ln (*SD*), TSI (*CHL*)=9.81 ln(*CHL*)+30.6, TSI (*TP*)=14.42 ln(*TP*)+4.15

| Table S3 Hydrochemical characteristics of the samples along the spatial-temporal analysis of the Valle de Bravo reservoir. | | | | | | | | | | | | | | |
| --- | --- | --- | --- | --- | --- | --- | --- | --- | --- | --- | --- | --- | --- | --- |
| Sample  Id | Hydrodinamic period | Water layer | Depth  (m) | Temp  (°C) | DO  (mg/L) | pH | SRSi  (Si-µM) | NH_4_^+^  (N-µM) | NO_2_^-^  (N-µM) | NO_3_^-^  (N-µM) | DIN  (N-µM) | TN  (N-µM) | SRP  (P-µM) | TP  (P-µM) |
| Sep.2018 |  |  |  |  |  |  |  |  |  |  |  |  |  |  |
| 2018.09.Z00 | Well stablished stratification | Epilimnion | 0 | 22.96 | 7.49 | 8.94 | 269.59 | 1.62 | 0.29 | 1.23 | 3.14 | 43.55 | 0.07 | 1.42 |
| 2018.09.Z01 | Well stablished stratification | Epilimnion | 1 | 22.7 | 7.8 | 9.0 | 267.7 | 1.6 | 0.1 | 0.3 | 1.9 | 41.0 | 0.1 | 0.7 |
| 2018.09.Z02 | Well stablished stratification | Epilimnion | 2 | 22.7 | 8.1 | 9.0 | 264.7 | 1.6 | 0.1 | 0.2 | 1.9 | 41.3 | 0.1 | 0.7 |
| 2018.09.Z04 | Well stablished stratification | Epilimnion | 4 | 22.3 | 8.0 | 9.0 | 261.4 | 1.1 | 0.1 | 0.2 | 1.3 | 40.6 | 0.2 | 0.7 |
| 2018.09.Z08 | Well stablished stratification | Metalimnion | 8 | 22.0 | 5.1 | 8.7 | 254.7 | 0.9 | 0.1 | 2.1 | 3.2 | 36.3 | 0.1 | 0.5 |
| 2018.09.Z12 | Well stablished stratification | Metalimnion | 12 | 21.2 | 0.3 | 8.1 | 255.3 | 26.1 | 0.2 | 0.4 | 26.7 | 55.4 | 0.4 | 0.9 |
| 2018.09.Z16 | Well stablished stratification | Hypolimnion | 16 | 20.9 | 0.2 | 7.8 | 251.3 | 45.3 | 0.1 | 0.3 | 45.7 | 80.6 | 1.3 | 1.7 |
| 2018.09.Z20 | Well stablished stratification | Hypolimnion | 20 | 20.7 | 0.2 | 7.6 | 250.3 | 53.1 | 0.1 | 0.1 | 53.4 | 94.9 | 1.7 | 2.3 |
| 2018.09.Z22 | Well stablished stratification | Hypolimnion | 24 | 20.7 | 0.1 | 7.5 | 247.7 | 58.7 | 0.1 | 0.1 | 58.9 | 100.0 | 2.0 | 2.6 |
| Dec.2018 |  |  |  |  |  |  |  |  |  |  |  |  |  |  |
| 2018.12.Z00 | Heterotrophic circulation | Epilimnion | 0 | 20.7 | 4.1 | 6.9 | 153.9 | 21.5 | 0.2 | 3.1 | 24.8 | 62.5 | 0.3 | 4.6 |
| 2018.12.Z01 | Heterotrophic circulation | Epilimnion | 1 | 20.5 | 4.0 | 6.9 | 157.8 | 19.5 | 0.2 | 2.1 | 21.8 | 71.0 | 0.2 | 4.4 |
| 2018.12.Z02 | Heterotrophic circulation | Epilimnion | 2 | 20.4 | 3.9 | 6.9 | 157.0 | 19.9 | 0.2 | 2.0 | 22.1 | 52.6 | 0.2 | 4.3 |
| 2018.12.Z04 | Heterotrophic circulation | Epilimnion | 4 | 20.2 | 3.7 | 7.0 | 157.1 | 19.8 | 0.2 | 2.0 | 22.0 | 59.1 | 0.4 | 4.4 |
| 2018.12.Z08 | Heterotrophic circulation | Metalimnion | 8 | 20.1 | 3.3 | 7.0 | 156.5 | 21.1 | 0.2 | 1.9 | 23.2 | 54.6 | 0.3 | 4.6 |
| 2018.12.Z12 | Heterotrophic circulation | Metalimnion | 12 | 20.1 | 3.1 | 7.0 | 158.2 | 21.1 | 0.2 | 1.8 | 23.1 | 48.0 | 0.3 | 4.6 |
| 2018.12.Z16 | Heterotrophic circulation | Hypolimnion | 16 | 20.1 | 2.3 | 7.0 | 152.2 | 22.6 | 0.3 | 1.9 | 24.7 | 57.9 | 0.3 | 4.7 |
| 2018.12.Z20 | Heterotrophic circulation | Hypolimnion | 20 | 20.0 | 2.5 | 7.0 | 161.1 | 22.5 | 0.2 | 1.6 | 24.3 | 55.9 | 0.3 | 4.6 |
| 2018.12.Z24 | Heterotrophic circulation | Hypolimnion | 24 | 20.0 | 2.1 | 7.0 | 149.4 | 24.0 | 0.3 | 2.0 | 26.2 | 56.2 | 0.4 | 4.6 |
| 2018.12.Z28 | Heterotrophic circulation | Hypolimnion | 28 | 19.8 | 1.2 | 7.0 | 152.9 | 26.0 | 0.2 | 2.2 | 28.4 | 63.1 | 0.4 | 5.0 |
| Apr.2019 |  |  |  |  |  |  |  |  |  |  |  |  |  |  |
| 2019.04.Z00 | Early Stratification | Epilimnion | 0 | 22.1 | 8.7 | 8.9 | 263.8 | 1.2 | 0.1 | 1.1 | 2.5 | 61.5 | 0.7 | 2.4 |
| 2019.04.Z01 | Early Stratification | Epilimnion | 1 | 21.5 | 8.9 | 8.9 | 261.8 | 0.8 | 0.1 | 0.6 | 1.5 | 65.6 | 0.6 | 3.1 |
| 2019.04.Z02 | Early Stratification | Epilimnion | 2 | 21.3 | 8.9 | 8.9 | 216.8 | 0.8 | 0.1 | 0.6 | 1.5 | 76.1 | 0.5 | 3.2 |
| 2019.04.Z04 | Early Stratification | Epilimnion | 4 | 21.0 | 8.4 | 9.0 | 248.3 | 1.1 | 0.1 | 0.8 | 1.9 | 78.4 | 0.5 | 1.9 |
| 2019.04.Z08 | Early Stratification | Metalimnion | 8 | 20.4 | 6.8 | 8.9 | 241.9 | 1.2 | 0.2 | 2.6 | 4.0 | 60.4 | 0.5 | 2.8 |
| 2019.04.Z12 | Early Stratification | Metalimnion | 12 | 19.5 | 2.7 | 8.4 | 244.9 | 1.6 | 0.4 | 12.6 | 14.7 | 54.6 | 0.7 | 1.7 |
| 2019.04.Z16 | Early Stratification | Hypolimnion | 16 | 19.0 | 0.2 | 8.0 | 256.5 | 3.5 | 0.6 | 17.6 | 21.7 | 44.3 | 1.0 | 2.6 |
| 2019.04.Z20 | Early Stratification | Hypolimnion | 20 | 18.8 | 0.1 | 7.8 | 258.4 | 7.6 | 0.7 | 14.7 | 23.0 | 64.1 | 1.0 | 5.2 |
| 2019.04.Z24 | Early Stratification | Hypolimnion | 24 | 18.8 | 0.1 | 7.6 | 223.6 | 9.8 | 0.5 | 11.0 | 21.3 | 77.9 | 1.2 | 3.9 |
| 2019.04.Z26 | Early Stratification | Hypolimnion | 26 | 18.7 | 0.1 | 7.6 | 234.0 | 10.1 | 0.5 | 11.3 | 21.9 | 69.9 | 0.5 | 2.2 |
| Sep.2019 |  |  |  |  |  |  |  |  |  |  |  |  |  |  |
| 2019.09.Z00 | Late stratification | Epilimnion | 0 | 23.0 | 8.5 | 9.2 | 408.6 | 1.9 | 0.1 | 1.6 | 3.6 | 46.9 | 0.2 | 0.7 |
| 2019.09.Z01 | Late stratification | Epilimnion | 1 | 22.9 | 8.2 | 9.2 | 399.5 | 1.1 | 0.1 | 1.6 | 2.8 | 55.4 | 0.3 | 1.2 |
| 2019.09.Z02 | Late stratification | Epilimnion | 2 | 22.8 | 7.8 | 9.2 | 417.2 | 0.7 | 0.1 | 1.3 | 2.1 | 54.0 | 0.3 | 1.1 |
| 2019.09.Z04 | Late stratification | Epilimnion | 4 | 22.6 | 7.2 | 9.2 | 419.7 | 0.3 | 0.1 | 0.7 | 1.1 | 53.3 | 0.2 | 1.2 |
| 2019.09.Z06 | Late stratification | Metalimnion | 6 | 22.5 | 6.8 | 9.1 | 436.2 | 0.7 | 0.1 | 1.7 | 2.4 | 53.4 | 0.4 | 1.2 |
| 2019.09.Z08 | Late stratification | Metalimnion | 8 | 22.3 | 5.8 | 9.1 | 426.4 | 0.5 | 0.1 | 0.8 | 1.4 | 53.5 | 0.1 | 0.9 |
| 2019.09.Z10 | Late stratification | Metalimnion | 10 | 22.1 | 3.5 | 8.9 | 422.5 | 5.9 | 0.2 | 1.7 | 7.7 | 53.9 | 0.2 | 0.7 |
| 2019.09.Z12 | Late stratification | Metalimnion | 12 | 21.9 | 1.3 | 8.6 | 418.5 | 11.2 | 0.4 | 2.5 | 14.1 | 54.2 | 0.2 | 0.5 |
| 2019.09.Z16 | Late stratification | Hypolimnion | 16 | 21.1 | 0.1 | 8.2 | 434.0 | 33.6 | 0.1 | 1.1 | 34.7 | 64.4 | 0.8 | 1.0 |
| 2019.09.Z20 | Late stratification | Hypolimnion | 20 | 21.0 | 0.2 | 7.9 | 421.8 | 49.3 | 0.0 | 0.6 | 49.9 | 50.2 | 1.4 | 1.7 |
| 2019.09.Z24 | Late stratification | Hypolimnion | 24 | 20.9 | 0.1 | 7.7 | 430.0 | 36.7 | 0.3 | 2.0 | 39.1 | 60.0 | 1.0 | 1.6 |
| 2019.09.Z28 | Late stratification | Hypolimnion | 28 | 20.8 | 0.1 | 7.7 | 421.7 | 54.9 | 0.2 | 0.3 | 55.4 | 87.9 | 1.6 | 3.7 |
| Jan.2020 |  |  |  |  |  |  |  |  |  |  |  |  |  |  |
| 2020.01.Z00 | Autotrophic circulation | Epilimnion | 0 | 20.0 | 5.6 | 7.4 | 307.0 | 1.2 | 0.2 | 14.6 | 16.0 | 46.9 | 0.1 | 1.5 |
| 2020.01.Z01 | Autotrophic circulation | Epilimnion | 1 | 19.6 | 5.7 | 7.3 | 284.1 | 0.6 | 0.1 | 12.8 | 13.6 | 55.4 | 0.1 | 1.8 |
| 2020.01.Z02 | Autotrophic circulation | Epilimnion | 2 | 19.5 | 5.7 | 7.2 | 334.9 | 0.8 | 0.1 | 14.5 | 15.4 | 54.0 | 0.1 | 1.9 |
| 2020.01.Z04 | Autotrophic circulation | Epilimnion | 4 | 19.3 | 5.5 | 7.0 | 325.4 | 0.6 | 0.1 | 14.4 | 15.1 | 53.3 | 0.1 | 1.3 |
| 2020.01.Z06 | Autotrophic circulation | Metalimnion | 6 | 19.3 | 5.2 | 7.1 | 316.8 | 0.6 | 0.1 | 12.1 | 12.7 | 53.4 | 0.1 | 0.8 |
| 2020.01.Z08 | Autotrophic circulation | Metalimnion | 8 | 19.3 | 5.1 | 7.4 | 381.0 | 0.5 | 0.1 | 15.2 | 15.9 | 53.5 | 0.1 | 2.4 |
| 2020.01.Z12 | Autotrophic circulation | Metalimnion | 12 | 19.3 | 5.1 | 7.7 | 312.7 | 3.0 | 0.1 | 14.2 | 17.3 | 54.2 | 0.1 | 1.4 |
| 2020.01.Z16 | Autotrophic circulation | Hypolimnion | 16 | 19.3 | 5.1 | 8.0 | 294.4 | 1.2 | 0.2 | 15.4 | 16.9 | 64.4 | 0.1 | 1.6 |
| 2018.08.Z20 | Autotrophic circulation | Hypolimnion | 20 | 19.3 | 5.1 | 8.2 | 320.5 | 1.0 | 0.1 | 11.8 | 12.8 | 50.2 | 0.1 | 2.3 |
| 2018.08.Z24 | Autotrophic circulation | Hypolimnion | 24 | 19.3 | 5.1 | 8.3 | 280.4 | 0.8 | 0.1 | 12.7 | 13.6 | 50.5 | 0.1 | 1.2 |
| 2018.08.Z27 | Autotrophic circulation | Hypolimnion | 27 | 19.3 | 5.1 | 8.2 | 321.8 | 0.9 | 0.1 | 11.5 | 12.5 | 50.5 | 0.1 | 2.1 |


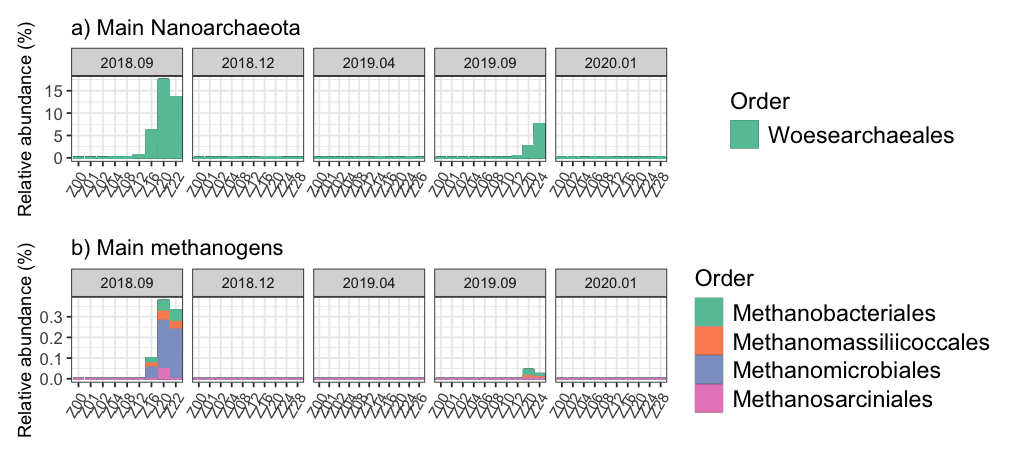


**Fig. S4** Relative abundance of the a) main Nanoarchaeota and b) main methanogens, along the water column at different sampling times.

| *Compartment*  Parameter | *r^2^* | *Pr*(>*r*) | Significance |
| --- | --- | --- | --- |
| *Water column* | | | |
| Depth | 0.416 | 0.001 | *** |
| Temperature | 0.5826 | 0.001 | *** |
| DO | 0.6987 | 0.001 | *** |
| pH | 0.2009 | 0.005 | ** |
| SRSi | 0.2553 | 0.003 | ** |
| NH_4_^+^-N | 0.6291 | 0.001 | *** |
| NO_2_^-^-N | 0.6118 | 0.001 | *** |
| NO_3_^-^-N | 0.4134 | 0.001 | *** |
| DIN | 0.6558 | 0.001 | *** |
| TN | 0.2901 | 0.001 | *** |
| SRP | 0.7669 | 0.001 | *** |
| TP | 0.2352 | 0.005 | ** |
| ^†^ Significance codes: . *p*-value < 0.10, **p*-value < 0.05, ***p*-value < 0.01, ****p*-value < 0.001 | | | |

**Fig. S5** CCA analyses results, showing the values of adjusted canonical coefficients (r2) and level of significance (p-value), for the physicochemical characteristics in the prokaryotic assemblages during the studied hydrodynamic periods. Below, the table with the numeric data can be observed.

| *Compartment*  Parameter | *r^2^* | *Pr*(>*r*) | Significance |
| --- | --- | --- | --- |
| *Water column* | | | |
| Depth | 0.8668 | 0.001 | *** |
| Temperature | 0.5927 | 0.001 | *** |
| DO | 0.7121 | 0.001 | *** |
| pH | 0.4846 | 0.001 | *** |
| SRSi | 0.4773 | 0.001 | *** |
| NH_4_^+^-N | 0.7769 | 0.001 | *** |
| NO_2_^-^-N | 0.1767 | 0.091 |  |
| NO_3_^-^-N | 0.2233 | 0.044 | * |
| DIN | 0.7671 | 0.001 | *** |
| TN | 0.3497 | 0.014 | * |
| SRP | 0.7956 | 0.001 | *** |
| TP | 0.5455 | 0.001 | *** |
| ^†^ Significance codes: . *p*-value < 0.10, **p*-value < 0.05, ***p*-value < 0.01, ****p*-value < 0.001 | | | |

**Fig. S6** CCA analyses results, showing the values of adjusted canonical coefficients (*r^2^*) and level of significance (*p*-value), for the physicochemical characteristics in the prokaryotic assemblages during the stratification periods. Below, the table with the numeric data can be observed.

**Fig. S7** Water depth changes during the sampled period in VB. The points in red represent those where the prokaryotic screening was done.


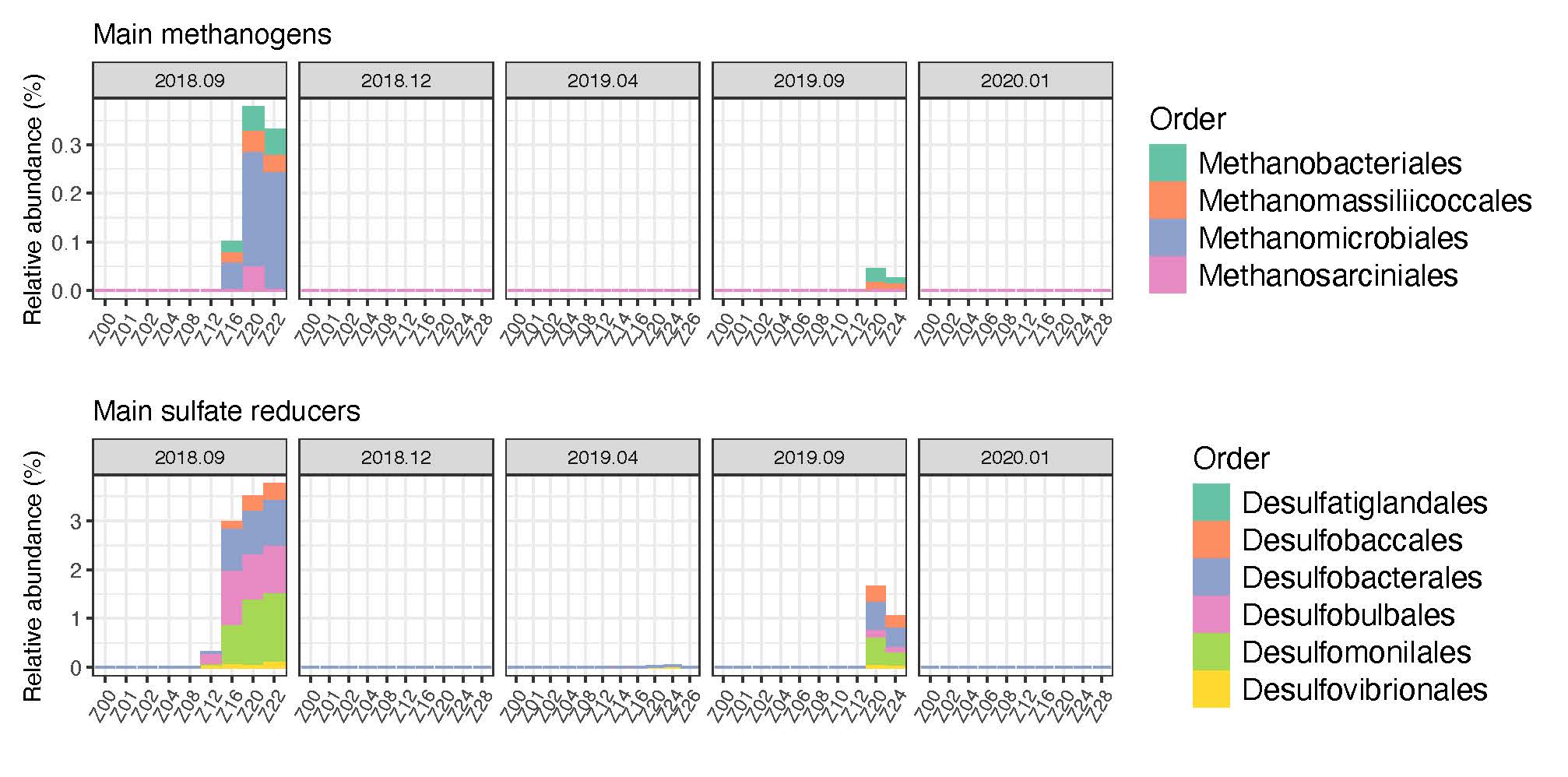


**Fig. S8** Relative abundance of the main sulphate reducers along the water column at different sampling times.


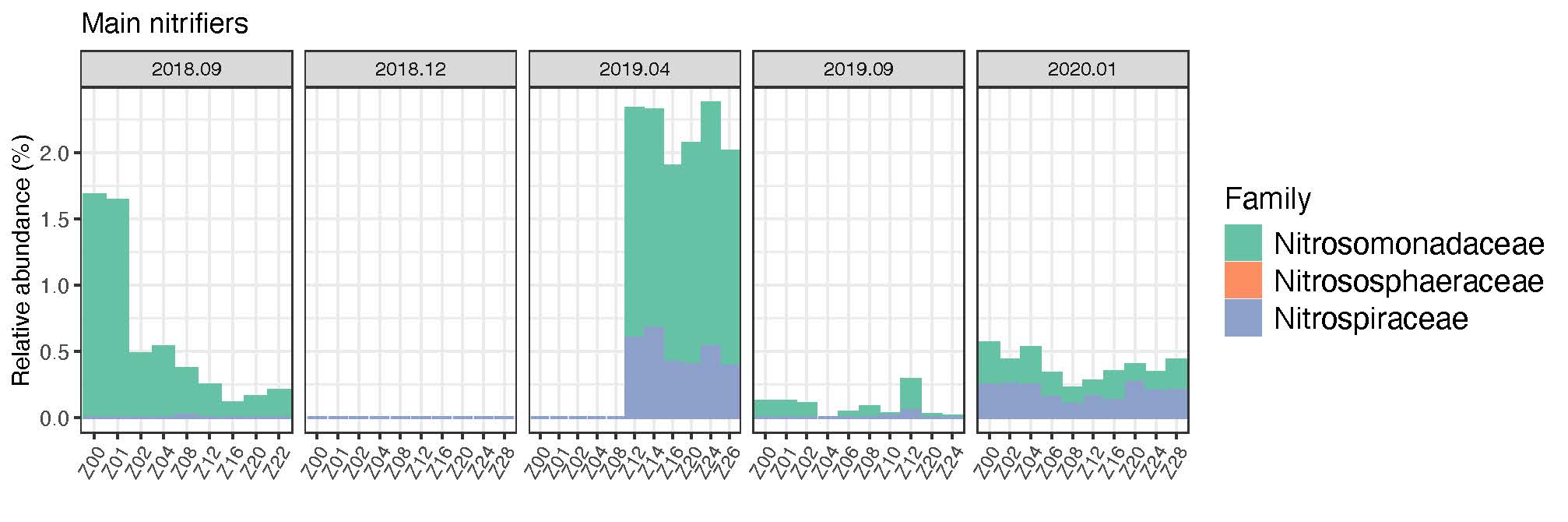


**Fig. S9** Relative abundance of the main nitrifiers along the water column at different sampling times. along the water column at different sampling times.

**Fig. S9** Vertical evolution of nutrients in the Valle de Bravo Reservoir. Vertical distribution of nitrate (NO_3_^-^, in purple), ammonia (NH_4_^+^, in yellow), both on the top axis, and nitrate (NO_2_^-^, gray) on the bottom axis, during the sampled months (September 2018, December 2018, April 2019, September 2019 and January 2020). The brown box represents the sediment layer, while the dashed blue lines indicate the beginning and the end of the oxycline.
